# miR-342 overexpression results in a synthetic lethal phenotype in *BRCA1*-mutant HCC1937 breast cancer cells

**DOI:** 10.18632/oncotarget.7617

**Published:** 2016-02-23

**Authors:** Elisabetta Crippa, Marco Folini, Marzia Pennati, Nadia Zaffaroni, Marco A. Pierotti, Manuela Gariboldi

**Affiliations:** ^1^ Department of Experimental Oncology and Molecular Medicine, Fondazione IRCCS Istituto Nazionale dei Tumori, Milan, Italy; ^2^ Molecular Genetics of Cancer, Fondazione Istituto FIRC di Oncologia Molecolare, Milan, Italy

**Keywords:** miR-342, BIRC6, Apollon/BRUCE, BRCA1-mutant, triple-negative breast cancer

## Abstract

Expression of miR-342 has been strongly correlated with estrogen receptor (ER) status in breast cancer, where it is highest in ER-positive and lowest in triple-negative tumors. We investigated the effects of miR-342 transfection in the triple-negative breast cancer cell lines MDA-MB-231 and HCC1937, the latter carrying a germ-line *BRCA1* mutation. Reconstitution of miR-342 led to caspase-dependent induction of apoptosis only in HCC1937 cells, while overexpression of wild-type BRCA1 in HCC1937 cells counteracted miR-342-mediated induction of apoptosis, suggesting that miR-342 overexpression and the lack of functional BRCA1 result in a synthetic lethal phenotype. Moreover, siRNA-mediated depletion of BRCA1 in MDA-MB-231 cells expressing the wild-type protein led to apoptosis upon transfection with miR-342. Using an *in silico* approach and a luciferase reporter system, we identified and functionally validated the Baculoviral IAP repeat-containing 6 gene (*BIRC6*), which encodes the anti-apoptotic factor Apollon/BRUCE, as a target of miR-342. In our model, *BIRC6* likely acts as a determinant of the miRNA-dependent induction of apoptosis in *BRCA1*-mutant HCC1937 cells. Together, our findings suggest a tumor-suppressive function of miR-342 that could be exploited in the treatment of a subset of *BRCA1*-mutant hereditary breast cancers.

## INTRODUCTION

Breast cancer (BCa) is the most frequent cancer diagnosed in women and the leading cause of death among females worldwide, with an estimated 1.7 million new cases and 500,000 deaths in 2012 [1]. BCa is a highly heterogeneous disease characterized by different levels of estrogen (ER) and progesterone (PR) receptors and epidermal growth factor receptor 2 (HER2). Based on the expression of these biopathological features, three major BCa subgroups have been identified: ER- and PR-positive, HER2-positive (carrying amplified HER2) and triple-negative (TNBC, with no expression of hormone receptors and no HER2 amplification) [2]. Generally, patients with TNBC have a poorer outcome than other subgroups [3, 4].

MicroRNAs (miRNAs) are small non-coding RNAs that negatively regulate gene expression through a mechanism that leads to mRNA degradation or repression of translation [5]. miRNAs are involved in many biological processes, such as the cell cycle, cellular differentiation, proliferation and apoptosis, where they can regulate complex gene pathways [6, 7]. Each tissue presents a specific miRNA expression pattern, which is deregulated when cancer develops. Depending on their target genes or the pathways involved, miRNAs can act both as tumor suppressors and as oncogenes (oncomiRs) [8–10].

Deregulated oncomiRs and tumor-suppressor miRNAs have been found to be involved in the development and progression of BCa. In this context, oncomiRs, such as miR-21, miR-155, miR-10b, miR-27a and miR-9, have been associated with BCa cell proliferation and development of metastasis, whereas tumor-suppressor miRNAs, including members of the let-7 family, miR-145, miR-200, miR-205, miR-335 and miR-19a, have been linked to the epithelial-mesenchymal transition and cell stemness maintenance in BCa [11]. miRNAs whose expression levels may help in distinguishing among BCa subgroups have also been identified [12]. In light of their pivotal role in gene regulation and their deregulated levels in cancer, miRNAs are now considered as very promising targets or tools for the development of novel anti-cancer therapeutic approaches [13].

We previously reported that expression of miR-342 was higher in ER-positive tumors and barely detectable in TNBC [14]. We also showed that miR-342-mediated reduction of ID4 protein expression resulted in increased BRCA1 expression in an *in vitro* model of TNBC [14], suggesting that the loss/low levels of the miRNA may account for the reduced expression of BRCA1 frequently observed in wild-type BRCA1 BCa. To further investigate the functional role of miR-342 in BCa, we transfected two TNBC cell lines with a synthetic precursor of the miRNA. The ectopic reconstitution of miR-342 expression levels in HCC1937 BCa cells, which harbor a homozygous loss-of-function *BRCA1* mutation [15], resulted in the induction of apoptosis as a consequence of reduced levels of the anti-apoptotic protein Apollon/BRUCE [16, 17], which we proved to be a direct miR-342 target. The protein, encoded by the *BIRC6* gene and a member of the inhibitors of apoptosis protein (IAP) family, plays a critical role in counteracting apoptosis by inhibiting caspases as well as SMAC/Diablo [16, 17].

Overall, our data show that miR-342 expression synergizes with the loss of functional BRCA1 in promoting apoptosis in HCC1937 TNBC cells, identifying miR-342 reconstitution as a promising avenue to therapy in patients with BRCA1-mutant hereditary BCa.

## RESULTS

### miR-342 reconstitution activates the intrinsic apoptotic pathway in HCC1937 cells

**Figure 1:**
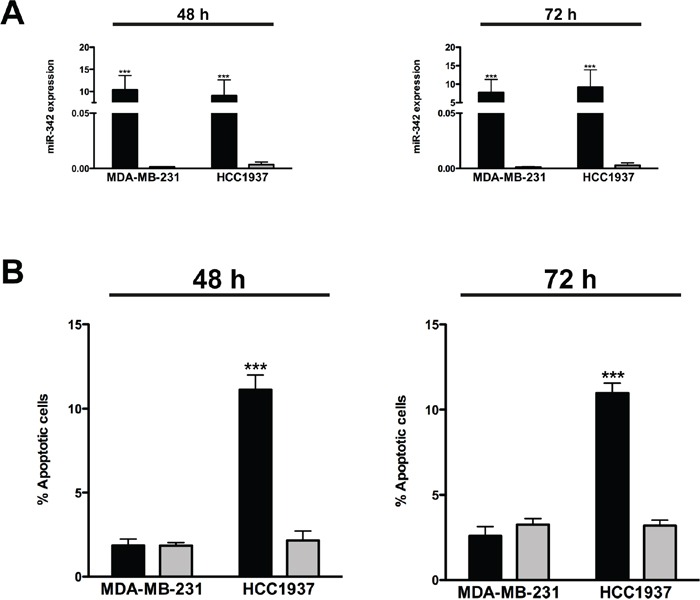
miR-342 overexpression induces apoptosis in *BRCA1*-mutant breast cancer cells MDA-MB-231 and HCC1937 cells were transfected with pre-miR-342 (black) or pre-miR-negative control (grey) and collected 48 and 72 hours later. **A.** qRT-PCR data showing the expression levels of mature miR-342 in breast cancer cells after transfection. Data are expressed as 2^−ΔCt^ (delta cycle threshold), which is directly related to the miRNA expression levels in each sample, and represent mean ± SD from three independent determinations. ***P<0.001 *vs* scramble. **B.** Quantification of data obtained by TUNEL assay upon miR-342 overexpression. Data are mean ± SD from three independent determinations. ***P<0.001 *vs* scramble.

**Figure 2:**
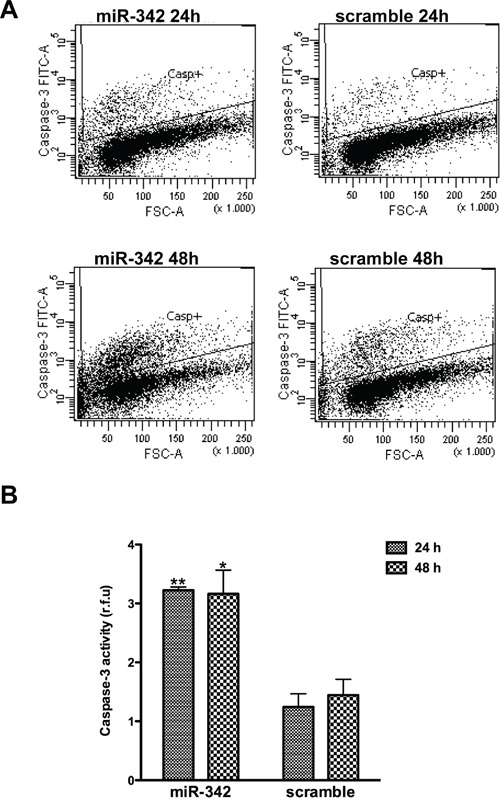
miR-342 overexpression activates the intrinsic pathway of apoptosis in HCC1937 cells HCC1937 cells transfected with pre-miR-342 or with pre-miR-negative control were collected 24 and 48 hours after transfection. **A.** Representative flow cytometric detection of cells containing active caspase-3 from three independent experiments. **B.** Caspase-3 catalytic activity, based on hydrolysis of a specific fluorogenic substrate. Data are given as relative fluorescence units (r.f.u.) and represent mean ± SD from three independent determinations. *P<0.05 and **P<0.01 *vs* scramble.

Based on evidence that miR-342 induces apoptosis in cancer cells [18], we assessed whether overexpression of the miRNA had a similar effect in TNBC cell lines MDA-MB-231 and HCC1937, which are characterized by markedly lower miR-342 expression levels compared to ER-positive cells [14]. qRT-PCR analysis revealed remarkably higher levels of mature miR-342 in both cell lines upon transfection with pre-miR-342 precursor molecule as compared to levels in cells transfected with a pre-miR negative control oligomer (Figure 1A). However, cell viability, as measured by MTT assay, was significantly reduced only in miR-342-transfected HCC1937 cells (Supplementary Figure 1) in association with the induction of apoptosis. Indeed, TUNEL assay showed that the percentage of apoptotic cells was 5- and 4-fold higher (P<0.001) after a 48- and 72-hours transfection, respectively, of HCC1937 cells with pre-miR-342 compared to cells transfected with pre-miR negative control (Figure 1B). By contrast, the percentage of apoptotic cells did not differ appreciably between miR-342-expressing MDA-MB-231 cells and negative control-transfected cells (Figure 1B). These findings were consistent with results of flow cytometric analysis of cleaved caspase-3 (Figure 2A) and with the marked induction of apoptosis in pre-miR-342-transfected HCC1937 cells as a function of caspase-3 catalytic activity (Figure 2B). Moreover, caspase-9 catalytic activity was significantly increased in HCC1937 cells transfected with the miRNA precursor (Supplementary Figure 2), suggesting that miR-342 overexpression contributes to activating the intrinsic apoptotic pathway in these *BRCA1*-mutant cells.

### The *BRCA1*-mutant genetic background favors the miR-342-mediated apoptotic effect

**Figure 3:**
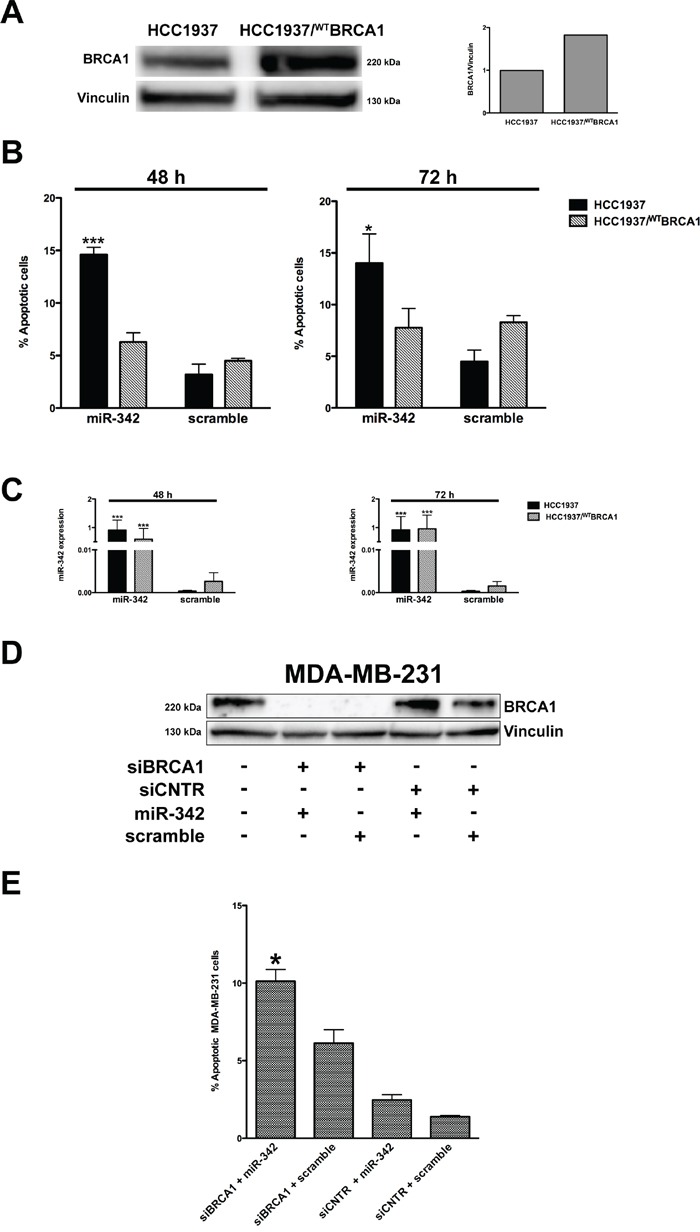
miR-342 induces apoptosis in a *BRCA1*-mutant context **A.** Representative Western blot from three independent experiments showing the expression of BRCA1 in HCC1937 cells and in the full-length *BRCA1* cDNA-transfected (HCC1937/^WT^BRCA1) cells. Vinculin was used as loading control. Data were quantified using Imagelab© software and normalized to vinculin. **B.** Quantification of data obtained by TUNEL assay 48 and 72 hours after transfection of pre-miR-342 or scramble in HCC1937 and HCC1937/^WT^BRCA1 cells. Data are mean ± SD from at least three independent determinations. *P<0.05 and ***P<0.001 *vs* scramble. **C.** qRT-PCR evaluation of expression levels of mature miR-342 in HCC1937 and HCC1937/^WT^BRCA1 cells after transfection. Data are reported as 2^−ΔCt^ (delta cycle threshold) value, which is directly related to the miRNA expression levels in each sample, and represent mean ± SD from at least three independent determinations. ***P<0.001 *vs.* scramble. **D.** A representative Western blot from three independent experiments showing the BRCA1 protein amounts in MDA-MB-231 cells as a function of the indicated transfections. Vinculin was used as protein loading control. **E.** Quantification of data obtained by TUNEL assay upon siRNA treatment of BRCA1 or of CNTR (control siRNA) followed 48 hours later by transfection of pre-miR-342 or scramble for 72 hours. Data are mean ± SD from at least three independent determinations. *P<0.05 *vs* siRNA BRCA1 + scramble.

To assess the possible role of the genetic background of *BRCA1*-mutant HCC1937 cells in the apoptosis-inducing capability of miR-342, we re-introduced the wild-type form of *BRCA1* in HCC1937 parental cells. Western blotting analyses showed an increase of BRCA1 protein abundance in a stable, G418-resistant transfected clone (HCC1937/^WT^BRCA1) (Figure 3A), indicating the effective restoration of the wild-type protein. TUNEL assay did not reveal an enhanced rate of apoptotic cell death in HCC1937/^WT^BRCA1 cells with respect to parental cells (Figure 3B), despite comparable levels of ectopically-reconstituted miR-342 (Figure 3C). This result corroborates the involvement of mutant BRCA1 in the miR-342-mediated apoptotic response and suggests that overexpression of miR-342 in the context of a mutant *BRCA1* genetic background results in a synthetic lethal phenotype [19]. Indeed, depletion of BRCA1 in MDA-MB-231 cells by an RNAi-mediated silencing approach led to a marked increase in the percentage of apoptotic cells upon transfection with the pre-miR-342 compared to BRCA1-depleted cells transfected with the pre-negative control (P=0.025) or to scramble-siRNA-transfected BRCA1-proficient cells, independently of restored miR-342 expression levels (Figure 3D and 3E).

### miR-342 targets the anti-apoptotic gene *BIRC6* in HCC1937 cells

**Figure 4:**
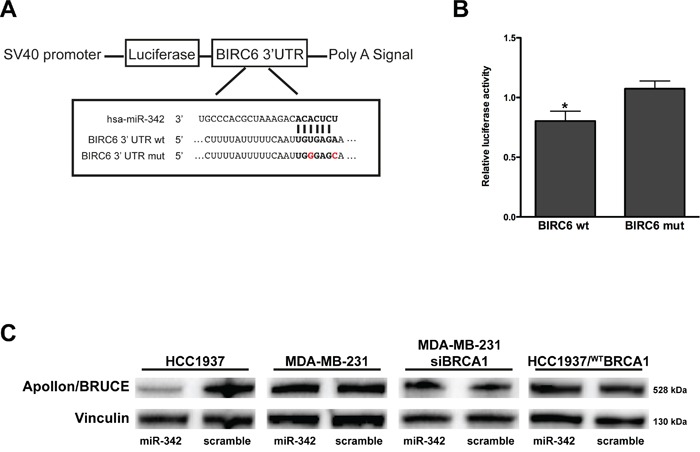
miR-342 targets *BIRC6* and down-modulates Apollon/BRUCE protein in HCC1937 cells **A.** Schematic representation of the interaction of miR-342 at nucleotides 204-210 of the wild-type (wt) and mutated (mut) BIRC6 3′ UTR cloned into pGL3 promoter vectors (pLuc–BIRC6 and pLuc-MUT-BIRC6). **B.** Quantification of relative luciferase activity (RLU) in 293T cells upon transfection with pre-miR-342 or scramble. Data are given as the ratio between luciferase activity detected in pre-miR-342 *vs* scramble-transfected cells and represent mean ± SD from at least three independent determinations. *P<0.05. **C.** Representative Western blot from three independent experiments showing Apollon/BRUCE protein expression after transfection of pre-miR-342 or of a scramble in HCC1937, MDA-MB-231, MDA-MB-231 siRNA BRCA1, and HCC1937/^WT^BRCA1 cells. Vinculin was used as loading control.

To further investigate the miR-342-mediated apoptotic effect, we focused on the *BIRC6* gene [20], which we found listed as a miR-342 predicted target in at least two public target prediction databases (TargetScan v6.2 and microRNA.org, released August 2010) and whose inhibition induces caspase-3-dependent apoptosis in BCa cells [21]. To functionally validate miR-342 binding to the 3′UTR of *BIRC6* and its potential inhibitory effect on the expression of the gene, we used a dual luciferase reporter system. Specifically, the 3′UTR region of *BIRC6* containing the predicted miR-342 binding site (Figure 4A) was cloned downstream of the *Firefly* luciferase gene and the resulting construct (pLuc-BIRC6) was transfected into 293T cells in the presence of the pre-miR-342 or the scramble. A vector containing a mutation in the putative miR-342 3′UTR binding site of *BIRC6* (pLuc-MUT-BIRC6) was used as control. As shown in Figure 4B, restoration of miR-342 expression levels resulted in a ~20% reduction in luciferase activity (expressed as the ratio of enzyme activity in pre-miR-342- to scramble precursor-transfected cells) in wild-type pLuc-BIRC6-transfected cells compared with cells expressing pLuc-MUT-BIRC6 (P=0.017). Evaluation of endogenous expression levels of the Apollon/BRUCE protein encoded by *BIRC6* in TNBC cell lines transfected with pre-miR-342 (Figure 4C) revealed a pronounced reduction of Apollon/BRUCE protein abundance in HCC1937 cells, whereas no significant differences in Apollon/BRUCE protein amounts were observed in miR-342-transfected MDA-MB-231 cells carrying wild-type *BRCA1*. This observation suggests that the down-modulation of Apollon/BRUCE by miR-342 and the consequent miRNA-dependent induction of apoptosis occur only in the presence of mutant *BRCA1*. In fact, RNAi-mediated depletion of *BRCA1* in MDA-MB-231 cells did not result in the down-modulation of Apollon/BRUCE expression (Figure 4C), although these cells underwent apoptosis upon restoration of miR-342 (Figure 3E). This finding rules out the possibility that wild-type BRCA1 acts as an endogenous miR-342 sponge. Although we cannot exclude that the lack of changes in Apollon/BRUCE expression levels upon transfection with pre-miR-342 in MDA-MB-231 cells reflects changes in the 3′UTR of the gene, Apollon/BRUCE levels did not change in HCC1937 cells ectopically expressing full-length BRCA1 (Figure 4C). Together, these findings suggest that the wild-type form of BRCA1 prevents recognition of *BIRC6* by miR-342 and that mutant *BRCA1* favors the miR-342-dependent down-modulation of Apollon/BRUCE.

**Figure 5:**
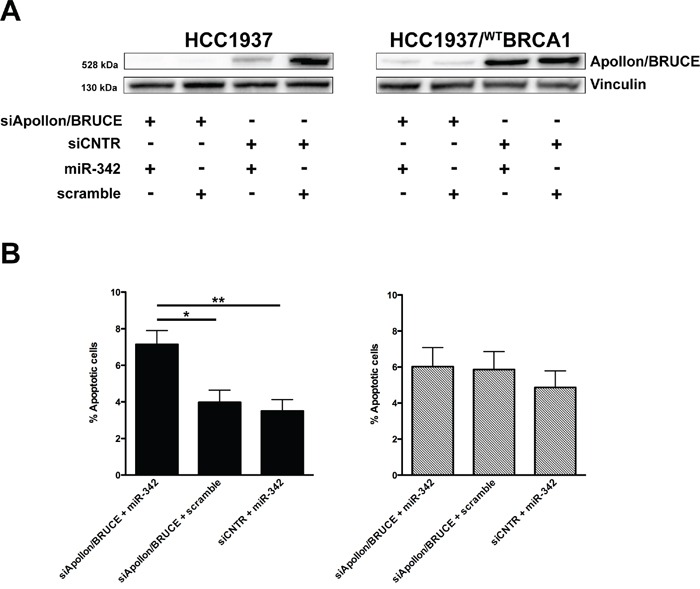
Apollon/BRUCE silencing contributes to miR-342-mediated induction of apoptosis in *BRCA1*-mutant HCC1937 cells **A.** Representative Western blot from three independent experiments showing Apollon/BRUCE protein amounts in HCC1937 and HCC1937/^WT^BRCA1 cells as a function of the indicated transfections. Vinculin was used as loading control. **B.** Quantification of data obtained by TUNEL assay upon miR-342 overexpression in HCC1937 (left) and HCC1937/^WT^BRCA1 (right) cells. Data are mean ± SD from three independent determinations. *P<0.05 and **P<0.01

To confirm that the effect of miR-342 on *BIRC6* depends on the presence of mutated *BRCA1*, we silenced *BIRC6* in both HCC1937 and HCC1937/^wt^BRCA1 cells (Figure 5A) and evaluated the rate of apoptosis after miR-342 reconstitution. TUNEL assay revealed an enhanced apoptotic response following the concomitant depletion of *BIRC6* and the overexpression of miR-342 compared to the single genetic manipulations in HCC1937 cells with respect to HCC1937/^wt^BRCA1 cells (Figure 5B). This result confirmed that the pro-apoptotic function of miR-342 is strictly dependent on the presence of mutated *BRCA1* and suggested that additional miRNA targets may be involved in the establishment of the synthetic lethal phenotype.

## DISCUSSION

Available literature data indicate that miR-342 expression is deregulated in different tumor types. Its down-modulation, which is suggestive of an oncosuppressor function, has been observed in hematopoietic and lymphoid malignancies, including early T-cell precursor acute lymphoblastic leukemia [22] and Sézary syndrome (a rare and aggressive leukemic variant of cutaneous T-cell lymphoma) [23] and in acute promyelocytic leukemia (APL) blasts compared with normal promyelocytes [24]. The miRNA was also found down-modulated in solid tumors, such as lung, gastric and colorectal cancers [18, 25–27]. However, little is known about the specific role of miR-342 in cancer. Reconstitution of miR-342 has been reported to induce apoptosis in HT29 colorectal cancer cells [18] but not in APL [28]. More recent studies reported the ability of this miRNA to counteract cell proliferation and invasion through inhibition of DNA methyltransferase 1 in SW480 colorectal cancer cells [27] and inhibition of the transcription factor FOXM1 in cervical cancer cells [29]. Moreover, high miR-342 expression levels have been associated with a better response to therapies in different tumor types. For example, upregulation of miR-342 levels was observed in APL patients who responded to all-trans acid retinoic-based therapy [24, 28, 30], and elevated levels of miR-342 have been associated with a prolonged time to progression after chemotherapy in gastric cancer patients [26]. In BCa, miR-342 expression levels have been correlated with those of ER, which is higher in tumors with favorable prognosis [12]. In this context, we previously showed that miR-342 is upregulated in ER-positive BCa and that its overexpression in a TNBC cell line increases BRCA1 levels through the down-modulation of ID4 [14]. Finally, down-modulation of miR-342 was described in BCa models resistant to Tamoxifen, one of the most widely used adjuvant therapies for this tumor type [31–33]. Our present results support the tumor-suppressor function of miR-342 observed in other cancers [18], since overexpression of this miRNA induced apoptosis in a BRCA1-mutant TNBC model.

BRCA1 not only plays a critical role in multiple cellular processes, including cell cycle progression, genomic integrity, development and apoptosis [34], but also can induce or repress apoptosis, depending on the type of chemotherapeutic drug used [35]. In fact, BRCA1 mediates sensitivity to apoptosis induced by anti-microtubule agents, while *BRCA1*-mutant cells, defective in DNA repair, are more sensitive to DNA-damaging agents that can trigger intracellular cell signals leading to activation of the intrinsic apoptotic pathway [36]. miRNAs can control *BRCA1* through different mechanisms, either involving the canonical binding to specific target regions in the 3′ UTR of the gene, as shown for miR-146, miR-182 and miR-638 [37–39], or by regulating genes that control BRCA1 expression, such as the transcription factors ID4 and E2F6 or the methyltransferase DNMT1 targeted by miR-342 and miR-185, respectively [14, 40].

Here, we show that overexpression of miR-342 in a mutant BRCA1 background can result in cell death by triggering apoptosis, a finding supported by the abrogation of the pro-apoptotic effect of miR-342 upon reconstitution of wild-type *BRCA1* expression levels in the HCC1937 *BRCA1*-mutant cells. Similar observations have been described for the inhibitors of poly(ADP) polymerase (PARP) in *BRCA1/2*-mutant BCa [41]. The involvement of miRNAs in synthetic lethality [19, 42] has also been recently described in retinoblastoma, where the inactivation of a miR-17-92 cluster has a synthetic lethal interaction with RB/p53 pathways, resulting in the suppression of tumor formation [43]. These data point to the possibility of developing new miRNA-based therapeutic approaches as a function of specific genetic backgrounds. As for miR-342, the observed biological effects attributable to synthetic lethality in a BRCA1-mutant context could be a promising strategy for treatment of the large proportion of BRCA1-mutant patients who do not benefit from therapies involving PARP inhibitors [44].

Our computational approach to searching for putative targets of miR-342 that could be involved in apoptosis led us to focus on the *BIRC6* gene, which encodes the IAP family member Apollon/BRUCE [16, 17]. We previously showed that Apollon/BRUCE silencing in BCa cells promoted apoptosis through the activation of caspase-3 and -9 in a p53 wild-type background [21]. In our TNBC model, overexpression of miR-342 induced apoptosis by activating the intrinsic apoptotic pathway and reducing Apollon/BRUCE protein levels only in the *BRCA1*-mutant cell line. Moreover, silencing of the *BRCA1* gene in MDA-MB-231 cells resulted in apoptosis after miR-342 introduction. Although the pro-apoptotic effect of miR-342 may be at least in part due to its ability to suppress expression of the anti-apoptotic factor Apollon/BRUCE, which we found to be a direct target of miR-342, the mechanism by which miR-342 induces apoptosis only in a specific *BRCA1*-mutant background remains unclear. In fact, despite efforts to generalize our observations, no appreciable effects on cell viability and BIRC6 expression in MDA-MB-436 and SUM149PT cells, which carry BRCA1 mutations different than that found in HCC1937 cells [45], were detected upon miR-342 reconstitution (Supplementary Figure 1 and data not shown). However, it should be noted that basal levels of endogenous miR-342 are higher in these two TNBC cell lines than in HCC1937 cells (Supplementary Figure 3), raising the possibility that the miR-342-mediated pro-apoptotic effects rarely occur in cells already accustomed to relatively high levels of endogenous miRNA. We also excluded a possible association of miR-342 with mutant *BRCA2*, the other gene that predisposes to hereditary BCa, since its reconstitution did not impair the viability of BRCA2-mutant HCC1599 TNBC cells (Supplementary Figure 1).

Although the effects of miR-342 reconstitution are restricted to a specific genetic background, the *BRCA1* mutation found in HCC1937 cells is one of the most common germ-line mutations in individuals of Ashkenazi Jewish ancestry and in Central and Eastern Europeans [46], such that the potential therapeutic application of miR-342 will benefit a high proportion of hereditary breast cancer patients. It also remains possible that miR-342 reconstitution contributes to improving the efficacy of conventional cancer chemotherapy (e.g., cyclophosphamide combined with methotrexate and fluorouracil, adriamycin, and taxane- or platinum-based therapies) as well as of PARP inhibitors. Further investigations *in vivo* are clearly warranted.

## MATERIALS AND METHODS

### Cell lines

MDA-MB-231 cells were obtained from ATCC (American Type Culture Collection, Rockville, MD, USA); HCC1937 cells from DSMZ (Deutsche Sammlung von Mikroorganismen und Zellkulturen, Braunscheweig, Germany); MDA-MB-436 cells from CLS (Cell Lines Service GmbH, Eppelheim, Germany); SUM149PT cells from Asterand (Detroit, MI, USA); HCC1599 cells from DSMZ; and 293T human embryonic kidney cells from ICLC (Interlab Cell Line Collection, Istituto Nazionale per la Ricerca sul Cancro, Genova, Italy). Cells were authenticated at each batch-freezing by STR profiling (StemElite ID System, Promega, Madison, WI, USA). MDA-MB-231 cells were grown in RPMI + 5% fetal bovine serum (FBS); HCC1937 in RPMI + 15% FBS; MDA-MB-436 in DMEM-Ham's F12 + 10 % FBS; SUM149PT in Ham's F12 + 10% FBS + 10 mM Hepes + 5 μg/ml human insulin+ 1 μg/ml hydrocortisone; HCC1599 in RPMI + 20% FBS; and 293T in DMEM + 10% FBS. All cell lines were maintained as a monolayer in a humidified 5% CO_2_ atmosphere at 37°C.

### Design and synthesis of small interfering (si) RNAs

Four different siRNAs targeting specific consensus sequences (AA(N19)TT) within the open reading frame of *BRCA1* mRNA (GeneBank accession no. NM_007294.3) were designed using a siRNA target finder tool (http://www.ambion.com) (Supplementary Table 1) and chemically synthesized (Eurofins MWG Operon, Ebersberg, Germany). A blast search was carried out to exclude any alignment with other sequences in the human genome. A siRNA (siGENOME non-targeting siRNA#1) consisting of a scrambled oligonucleotide sequence with no significant homology to any known human mRNA was used as control (ThermoScientific Dharmacon, Rockford, IL, USA). A pool of four different siRNAs targeting specific consensus sequences within *BIRC6* mRNA, as well as a control siRNA, were obtained as described [21].

### Transfection

Cells were seeded in 60-mm dishes and transfected the next day with pre-miR-342, pre-miR™ precursor molecule ID: PM12328 (mature sequence: UCUCACACAGAAAUCGACCCGU) (Ambion, Austin, TX, USA), pre-miR negative control (“scramble”), and pre-miR™ miRNA precursor molecule, negative control #1, ID: AM17110 (Ambion) at a final concentration of 50 nM using Lipofectamine RNAiMAX (Invitrogen, Carlsbad, CA, USA) according to the manufacturer's instructions. Cells were collected and analyzed at different intervals after transfection.

HCC1937 cells were transfected with a pcDNA 3.1 plasmid containing the full-length *BRCA1* gene as described [47]. The construct was kindly provided by Dr. M. Montagna, Istituto Oncologico Veneto, Padova, Italy.

Gene-specific and control siRNAs (25 nM for *BRCA1* and 50 nM for *BIRC6*) were transfected into breast cancer cells using Lipofectamine RNAiMAX (Invitrogen). After 48 hours, transfection with pre-miR precursor molecules (50 nM) was carried out and cells collected after 72 (siBRCA1) or 48 (siApollon/BRUCE) hours.

### Quantitative real-time polymerase chain reaction (qRT-PCR)

Total RNA was extracted from cell lines using Trizol (Life Technologies, Frederick, MD, USA) according to the manufacturer's protocol. For miRNA expression analysis, 30 ng of total RNA in a final volume of 15 μL were reverse-transcribed to cDNA using a high-capacity cDNA reverse transcription kit and miR-342 specific primer (Applied Biosystems, Foster City, CA, USA) according to the manufacturer's instructions. RT-qPCR was carried out using FAST chemistry (Applied Biosystems) with the manufacturer-provided miRNA-specific assay in the ABI PRISM 7900 HT real-time PCR system (Applied Biosystems). Expression values of miR-342 (002260) were normalized to RNU6B (001093). Data were analyzed using Sequence Detector software SDS 2.1.

### Cell viability (MTT) assay

Cells were plated at a density of 6 × 10^4^ in 12-well plates and, at 72 hours after transfection, incubated for 3 hours at 37°C in medium containing MTT (3-(4,5-dimethylthiazol-2-yl)-2,5-diphenyltetrazolium bromide 0.5 mg/ml, Sigma-Aldrich, St. Louis, MO, USA). Lysis buffer (10% SDS and 0.01 M HCl in water) was then added to each well to dissolve the formazan crystals. Absorbance was measured on a microplate reader (Infinite M200 TECAN) at a wavelength of 570 nm.

### Apoptosis analysis

TUNEL assay was performed using the *in situ* Cell Death Detection Kit Fluorescein (Roche, Mannheim, Germany). Cells were fixed with 2% formaldehyde in PBS for 20 minutes on ice and incubated with terminal deoxynucleotidyl transferase and label solution (fluorescein-dUTP) for 1 hour at 37°C. After washing with PBS containing 1% BSA, the presence of TUNEL-positive cells was assessed using a FACSCalibur flow cytometer (Becton-Dickinson, Franklin Lakes, NJ, USA).

The percentage of cells positive for cleaved caspase-3 was determined using a rabbit anti-human cleaved caspase-3 antibody (dilution 1:50, Cell Signaling Technology, Danvers, MA, USA) followed by staining with goat FITC-labeled antibody (Cell Signaling) and assessment by flow cytometry.

The catalytic activity of caspase-3 and caspase-9 was measured as the ability to cleave the specific substrates N-acetyl-Asp-Glu-Val-Asp-7-amino-4-methyl-coumarin (DEVD-AMC) and N-acetyl-Leu-Glu-His-Asp-AMC (LEHD-AMC) using the APOPCYTO/Caspase-3 and APOPCYTO/Caspase-9 Fluorometric Assay Kits (Medical & Biological Laboratories, Naka-ku Nagoya, Japan), respectively, according to the manufacturer's protocol. Hydrolysis of the specific substrate for each caspase was monitored by spectrofluorometry with 380-nm excitation and 460-nm emission filters.

### Dual luciferase assays

For luciferase reporter experiments, the 3′ UTR region of *BIRC6* gene containing the predicted miR-342 binding site (387 bp) and a variant obtained by introducing two point mutations (nucleotides 3 and 7) in the seed region were amplified and cloned into the XbaI site of the reporter plasmid pGL3 promoter (Promega, Madison, WI, USA) downstream of the *Firefly* luciferase gene. The new vectors were named pLuc-WT-BIRC6 and pLuc-MUT-BIRC6, respectively. The following primers were used to amplify specific fragments:

WT-BIRC6:

Forward5′-GCATATTCTAGACTTCGAAGCACAAGCCAAAT-3′, and

Reverse 5′-GCATATTCTAGAAATTCAGTGAAAAGTTGCTGACT-3′

MUT-BIRC6:

Forward 5′-GCATATTCTAGACTTCGAAGCACAAGCCAAAT-3′, and

Reverse 5′-GTTGAACATACCAATCAGTGGTGCTCCCAATTGAAAAATAAAA

GCACAAAAAAG-3′

For the reporter assays, 5 × 10^4^ 293T cells were seeded in 24-well plates, grown for 24 hours, and transfected with 0.1 μg of pLuc-WT-BIRC6 or pLuc-MUT-BIRC6 and 0.01 μg of *Renilla* luciferase pRLTK plasmid (Promega) together with 100 nM of pre-miR-342 or the scramble using Lipofectamine 2000 (Invitrogen), according to the manufacturer's instructions. After 24 hours, luciferase activity was measured using the Dual Luciferase Assay kit (Promega), according to the manufacturer's instructions. *Firefly* luciferase activity was normalized to that of *Renilla* as a control for transfection efficiency and reported as relative light units (RLUs).

### Western blotting

Western blotting analyses were carried out for Apollon//BRUCE and BRCA1 protein. After transfection with miR-342 precursor or negative control, MDA-MB-231, HCC1937 and HCC1937/^WT^BRCA1 cells were trypsinized and resuspended in 1X SDS sample buffer (62.5 mM Tris-HCl, pH 6.8, 2% w/v SDS, 10% glycerol, 50 mM DTT), supplemented with protease inhibitor cocktail (Calbiochem, San Diego, CA, USA).

Proteins were quantitated using the BCA protein assay (Thermo Scientific, Rockford, IL, USA). Protein extract (40 μg) was loaded on precast 3-8% NuPAGE Tris-acetate gels for the detection of Apollon/BRUCE and on a 7% polyacrylamide gel for the detection of BRCA1 and transferred to nitrocellulose membranes. The following primary antibodies were used: anti-Apollon/BRUCE (dilution 1:5000, Abcam, Cambridge, UK); anti-BRCA1 (dilution 1:800, Cell Signaling Technology); and anti-vinculin (dilution 1:5000, Sigma-Aldrich). Signals were detected using enhanced chemiluminescence (Thermo Scientific).

### Statistical analyses

Student's *t*-test was used to analyze differences between pre-miR negative control- and pre-miR-342-transfected cells with respect to qRT-PCR, rate of cell viability and of apoptosis, detection of cleaved caspase-3-positive cells, catalytic activity of caspase-3 and caspase-9, and luciferase activity. P values < 0.05 were considered statistically significant.

## SUPPLEMENTARY FIGURES AND TABLE


