# Supplementary material for: miR-342 overexpression results in a synthetic lethal phenotype in BRCA1-mutant HCC1937 breast cancer cells
Source: Oncotarget. 2016 Feb 23;7(14):18594–604. doi: 10.18632/oncotarget.7617 (PMC4951312; doi:10.18632/oncotarget.7617)
Supplement: Supplementary file 1 [file oncotarget-07-18594-s001.pdf]

SUPPLEMENTARY FIGURES AND TABLE

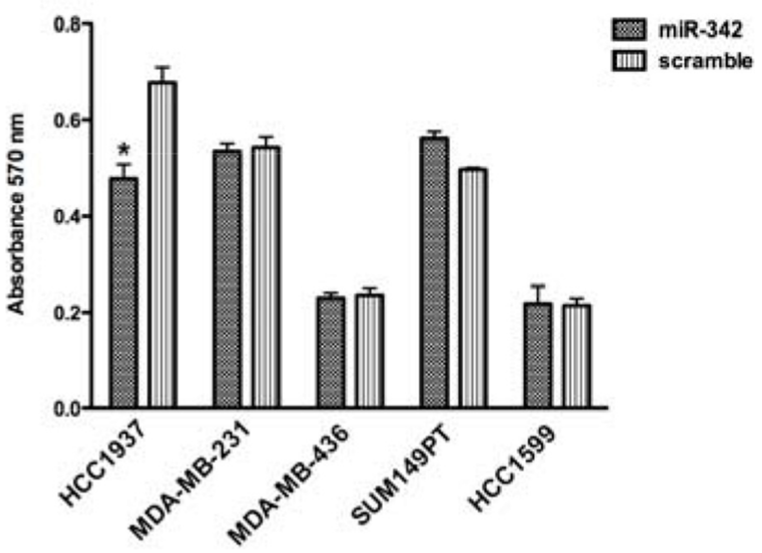

Supplementary Figure S1: miR-342 reduces cell viability in HCC1937 cells. MTT viability assay performed 72 hours after pre-miR-342 or pre-miR-negative control (scramble) transfection in the cell lines analyzed. Data represent mean ± SD from at least three independent determinations. *$P<0.05$ *vs.* scramble.

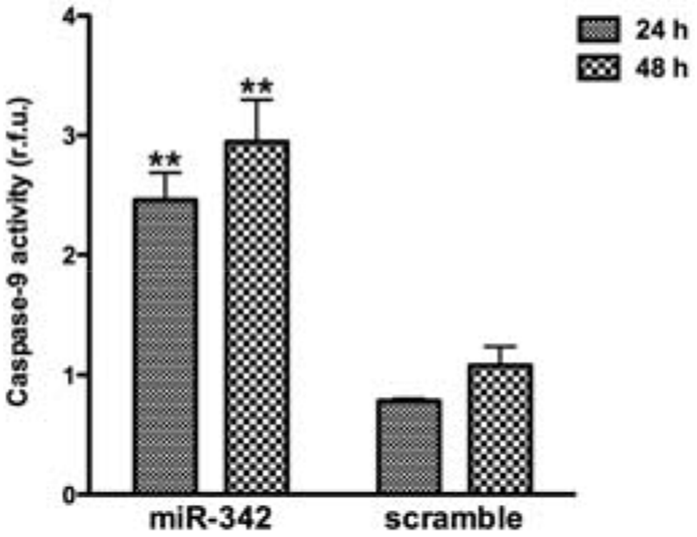

Supplementary Figure S2: miR-342 overexpression increases caspase-9 activity in HCC1937 breast cancer cells. Caspase-9 catalytic activity based on hydrolysis of a specific fluorogenic substrate. Data are given as relative fluorescence units (r.f.u.) and represent mean ± SD from at least three independent determinations. **P<0.01 *vs.* scramble.

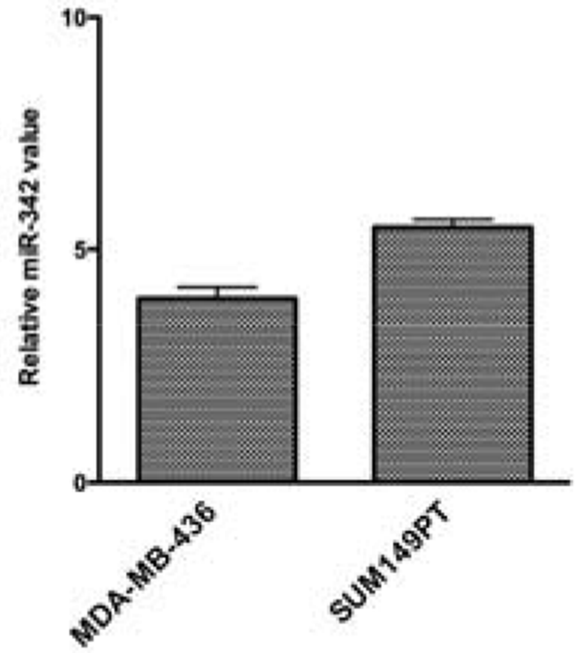

Supplementary Figure S3: MDA-MB-436 and SUM149PT cells express higher endogenous miR-342 than do HCC1937 cells. Relative quantification of miR-342 expression in MDA-MB-436 and SUM149PT cells as compared with that in HCC1937 cells. Data are expressed as $2^{-\Delta\Delta Ct}$ and represent mean ± SD from three independent determinations.

Supplementary Table S1: Sequences of siRNAs targeting *BRCA1* mRNA

Name	Sequence
***BRCA1* #1**	GAAGCCAGCUCAAGCAAUAdTdT<br>dTdTCUUCGGUCGAGUUCGUUAU
***BRCA1* #2**	GACGUCUGUCUACAUUGAAdTdT<br>dTdTCUGCAGACAGAUGUAACUU
***BRCA1* #5**	UAACUCUCCUGAACAUCUAdTdT<br>dTdTAUUGAGAGGACUUGUAGAU
***BRCA1* #6**	AGACGUCUGUCUACAUUGAdTdT<br>dTdTUCUGCAGACAGAUGUAACU
